# Global burden of urticaria: Insights from the 2016 Global Burden of Disease Study

**DOI:** 10.1016/j.jaad.2018.02.026

**Published:** 2018-09

**Authors:** Elena Maxim, Chante Aksut, Derrick Tsoi, Robert Dellavalle

**Affiliations:** a Michigan State College of Human Medicine, Southfield, Michigan; b Department of Dermatology, University of Colorado School of Medicine, Denver, Colorado; c Institute for Health Metrics and Evaluation, University of Washington, Seattle, Washington; d Dermatology Service, US Department of Veterans Affairs Eastern Colorado Health Care System, Denver, Colorado; e Department of Epidemiology, Colorado School of Public Health, Aurora, Colorado

*To the Editor:* Urticaria can be associated with significant functional impairment.^1^ While prevalence studies exist, few have explored the burden of urticaria worldwide, and to understand how a disease contributes to morbidity and mortality, measures beyond prevalence and incidence should be considered. The Global Burden of Disease (GBD) Study is a systematic, comprehensive effort to estimate the comparative magnitude of premature death and disability in >195 countries and 300 diseases.^2^ To our knowledge, this study is the first effort to present GBD results regarding the burden of urticaria by age, sex, and geography.

Complete details of GBD methodology are available elsewhere.^3^ Methods specific to the estimation of urticaria burden are presented here. A systematic review was performed on PubMed and Google Scholar to capture epidemiologic data for urticaria, including studies published during 1980-2014. Hospital outpatient and US claims data (using International Classification of Diseases, Tenth Revision, code L50) from 2000, 2010, and 2012 were also included. A Bayesian meta-regression tool, DisMod-MR 2.1, was used to model urticaria epidemiologic data points and estimate prevalence by age, sex, year, and country. Predictive covariates are used to create approximate estimates for countries lacking data. The GBD methods stratify disease-specific prevalence by severity level by assessing varying levels of itch, pain, and worry. Disability weights range from 0 (least disabling) to 1 (most disabling) and were derived from 4 population-based European surveys and an open-access web-based survey of 60,890 respondents.^3^ Disease burden is measured by disability-adjusted life years (DALYs), combining morbidity (years lost to disability) with mortality (life-years lost), such that 1 DALY is equivalent with 1 healthy life–year lost.^3^

**Fig 1:**
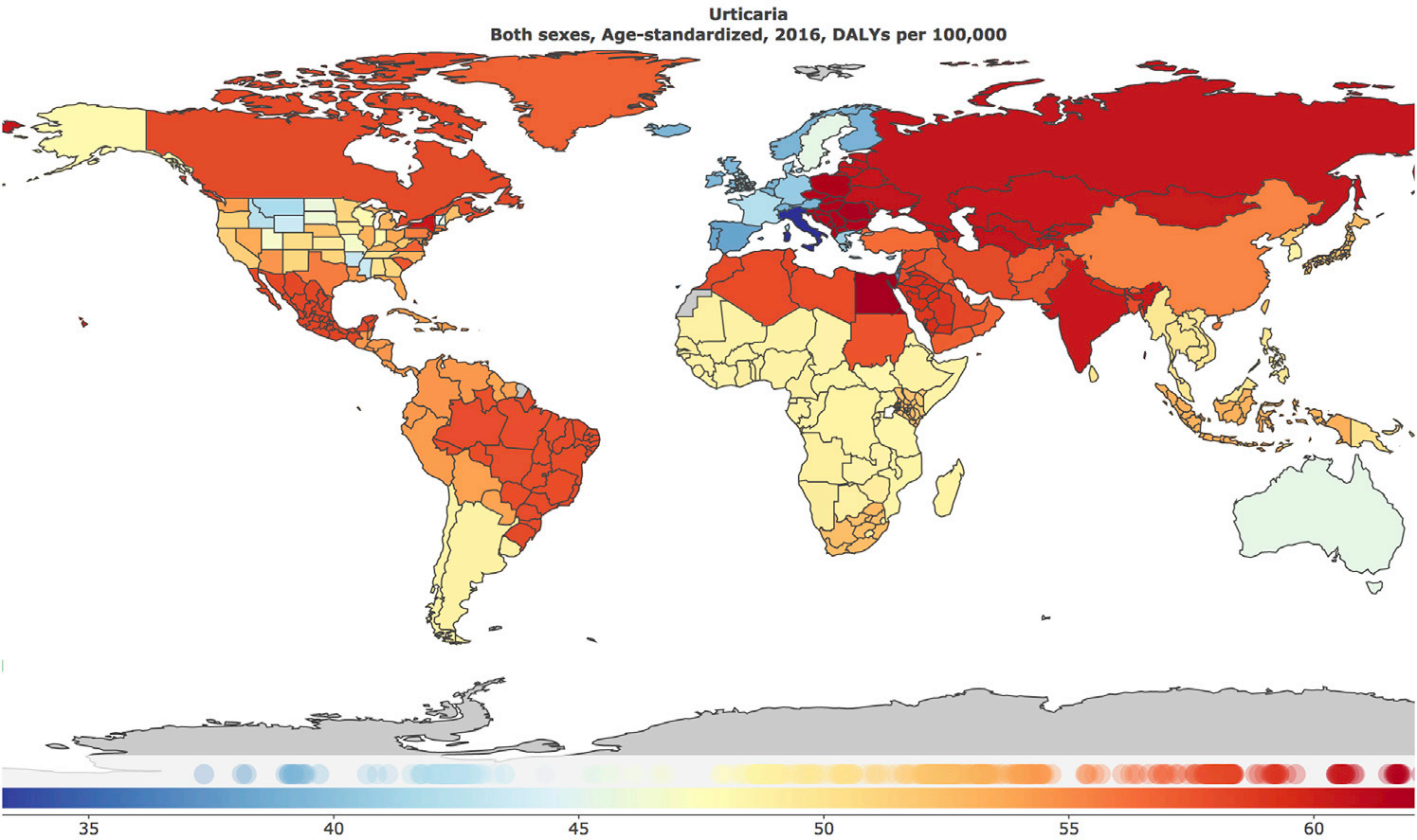
Geographic distribution of age-standardized DALYs per 100,000 population for urticaria in countries analyzed in the Global Burden of Disease Study. *DALY*, Disability-adjusted life years.

**Fig 2:**
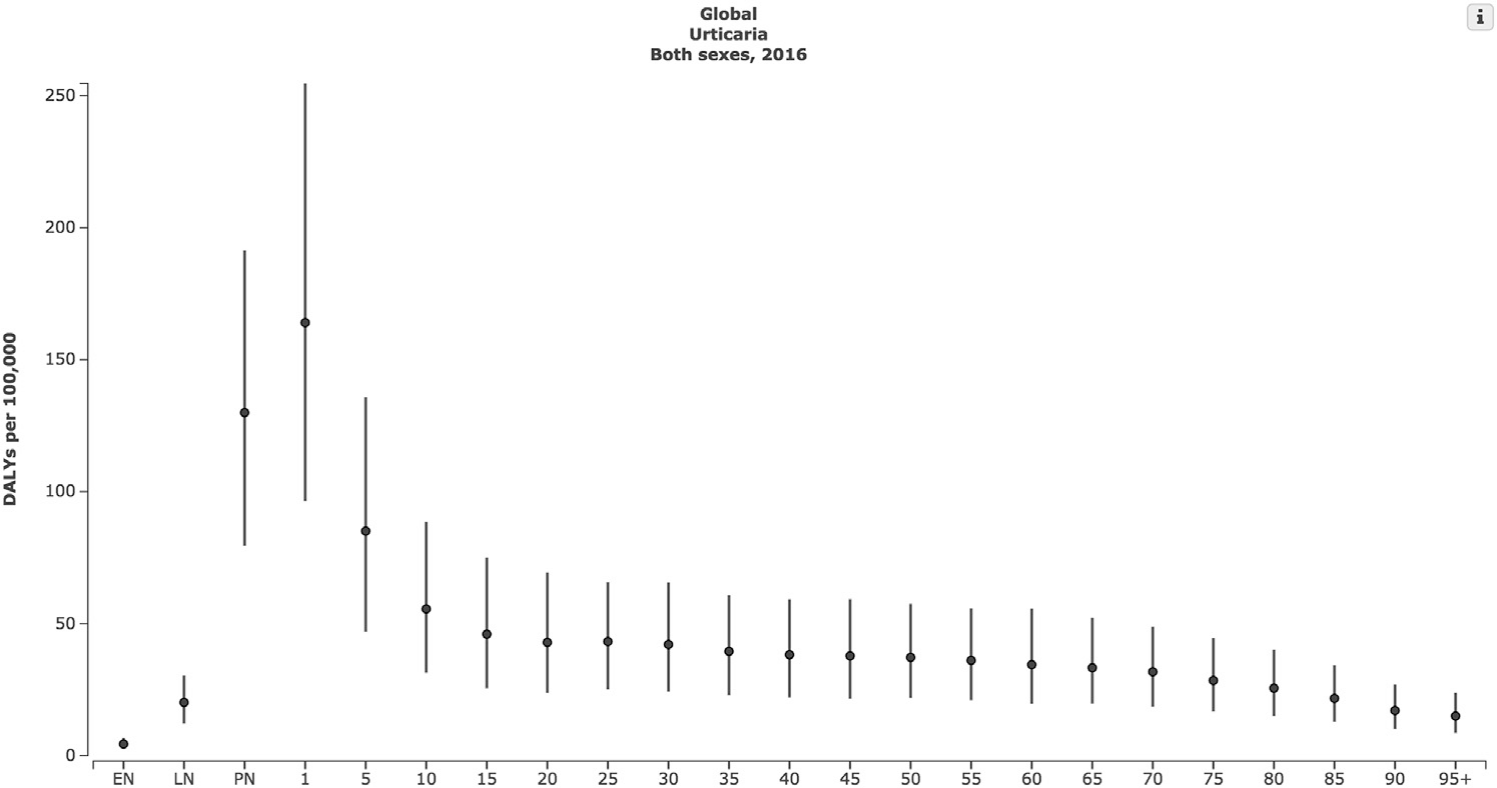
Total mean estimates of DALYs lost for urticaria with 95% confidence intervals by consecutive age category. Age categories shown reflect incidence at each year of life. *DALY*, Disability-adjusted life years; *EN*, early neonatal; *LN*, late neonatal; *PN*, postnatal period or the first 6 weeks after birth.

The global age-standardized DALY rate for urticaria is 55.49/100,000 population. Among skin disease, the global burden from urticaria in 2016 ranks only behind acne vulgaris (214/100,000), dermatitis (152/100,000), viral skin disease (80.02/100,000) and psoriasis (76/100,000).^2^ The 3 world regions with the greatest DALY rates for urticaria are Central Europe (61.8/100,000 population), Eastern Europe (60.7/100,000 population), and Central Asia (60.6/100,000 population). Fig 1 depicts the urticaria DALY rate per country. The age-standardized DALY rate for urticaria is 47.4/100,000 population and 62.5/100,000 population in male and female persons, respectively. Analysis of urticarial burden of disease by age group shows that children aged 1-4 years have the highest burden of disease (164.02/100,000 population), followed by postnatal infants (129.98/100,000 persons) and those aged 5-9 years (85.06/100,000 population). Fig 2 depicts the DALY rate for the consecutive age categories studied.

Limitations of the GBD Studies have been previously described.^4^ Although the GBD model relies on high-quality analysis and statistics, it is not a perfect prediction model, and the data do not differentiate between chronic and acute episodes of urticaria. Despite the limitations, the GBD data offer a solid base for further studies to build upon. Targeted therapy (anti-IgE) for urticaria has a potential to decreased overall morbidity associated with the disease.^5^ The data presented in this study highlight the variation in burden of disease by geography, sex, and age, which can be used to guide further research on targeted therapies, especially for populations heavily affected by urticaria.
